# Effect of a participative action intervention program on reducing mental retirement

**DOI:** 10.1186/s12889-019-6522-x

**Published:** 2019-02-14

**Authors:** Jenny J. J. M. Huijs, Irene L. D. Houtman, Toon W. Taris, Roland W. B. Blonk

**Affiliations:** 1 TNO (The Netherlands Organization for Applied Scientific Research), Leiden, Netherlands; 2 Dept. of Social, Health and Organizational Psychology, Utrecht University, Utrecht, Netherlands; 3 Dept. of Human Resource Studies, Tilburg School of Social and Behavioral Sciences, Tilburg, Netherlands; 4 Optentia Research, North-West University, Vanderbijlpark, South Africa; 5 Department Tranzo, North Wes University, Faculty of Humanities, Vanderbijlpark, South Africa

**Keywords:** Mental retirement, Participatory program, Tailor-made intervention, Effect study, Multilevel analysis

## Abstract

**Background:**

The present study aimed to investigate the effects of a stepwise, bottom-up participatory program with a tailor-made intervention process addressing the level of mental retirement in a sample of Dutch employees. Mental retirement refers to feelings of being disconnected from your work and your organization. Prevention of mental retirement is important since sustainable employability is becoming more important in today’s society due to the ageing of the working population and the changes in skills demands.

**Methods:**

This prospective cohort study with a one-year follow-up employs a sample of 683 employees of three organizations in The Netherlands, who filled out two questionnaires: at baseline and 1 year later. The dependent measure was mental retirement, which consists of three sub-concepts: developmental pro-activity, work engagement and perceived appreciation.

**Results:**

Multilevel analysis (*N* = 466) showed that employees who more actively participated in the intervention(s) had a small but statistically significant larger decrease in mental retirement at follow-up.

**Conclusions:**

The stepwise, bottom-up participatory program with a tailor-made intervention process shows a tendency to decrease the level of mental retirement in Dutch employees. However, the implementation of interventions could be further improved since it turned out to be very challenging to keep up participants’ commitment to the program. Future research should study the effectiveness of this program further with an improved study design (control group, multiple follow-ups, several data sources).

## Background

Sustainable employability – referring to employees’ capacities to function in work throughout their working life – has become an important issue in the last decades [1, 2]. Two major developments are responsible for this increased attention. First, due to technical developments, globalization and innovations, skills demands and the labor market are rapidly changing [3, 4]. Organizations need to be more flexible and adaptable, which requires other competencies from employees than before. This causes employees’ skills to become obsolete and endangers their sustainable employability. Second, the Dutch working population is rapidly ageing due to lower birthrates, a decline in mortality rates, and an increase in official pension ages [5, 6]. The average age of employees in the Netherlands was 36.2 years in 1990; in 2000 it had increased to 38.3 years, and in 2014 it had further increased to 41.9 years [7]. The effective retirement age has also increased considerably in the Netherlands. In the beginning of this century the effective retirement age was just below 61 years, in 2017 this had increased to almost 65 years [7].

These two developments lead to an increasing pressure for maintenance of physical, mental and cognitive abilities of the labor force to ensure that employees remain employable, stay healthy, motivated, competent and productive at least until the age or retirement [1, 2]. The prevention of *mental retirement* can play an important role in the maintenance of these abilities [8]. Employees who are mentally retired are disconnected from their work and from the organization. Compared to others, they invest less in their work, their employability and development, and they have gradually lost their connections with their job, their colleagues and the organization.

### The concept of mental retirement

Previous studies show that mental retirement consists of three factors: developmental pro-activity, work engagement and perceived appreciation [8, 9]. An indifference to learning and development can result in a decline, or even loss of skills, in skills obsolescence, a decrease in sustainable employment for both the internal labor market of an organization as well as the external labor market or it can even result in job loss [3, 4, 10]. Furthermore, engaged employees are better connected to their work, cope more effectively with job demands and perform better in their work [11, 12], while lower engagement is related to more sickness absence [13]. In addition, more perceived appreciation is linked to more job commitment [14, 15].

In the literature the concept of mental retirement has been mentioned before e.g. [16, 17]. However, in these earlier studies mental retirement has a rather different definition in which it is specifically linked to actual retirement and age. Mental retirement is defined for example as the cognitive decline that seems to occur after actual retirement [16]. This decline is caused by a lack of cognitive stimulation and mental exercise, which occurs when someone is retired as well as when an employee is still working but has the prospect of nearby early retirement. In another study mental retirement is defined as a decrease in work engagement for employees who are facing actual retirement [17]. However, this study finds no support for this relation. Another related concept that is also linked to age, is the ‘older worker identity’. This refers to the internalization of negative attitudes and beliefs regarding the older worker, mostly based on stereotypes (e.g. low motivation, resistance to change, inflexibility and lack of interest in learning) [18, 19]. This internalization can be caused by discrimination in career opportunities and feelings of deprivation in comparison to others. Older worker identity is positively related to full retirement and negatively to late retirement, job mobility and development opportunities on the job [19, 20].

In contrast to the concept of older worker identity and the definitions mentioned earlier on mental retirement, mental retirement is not necessarily connected to age since a previous study showed no differences in the level of mental retirement in different age groups [8]. Although studies show that older employees participate to a lesser degree in training and maintenance of their working skills [21–24], a study on lifelong learning in the Netherlands shows that there may be a trend shift over time with regard to training participation [5, 25]. In the past, training participation clearly declined with age, but data from 2010 indicates that training participation remains stable. In addition, studies have shown that the importance of meaningful work, development opportunities and being appreciated increases with age of retirement [26, 27].

Although mental retirement is a fairly new concept and more research is needed to study the predictors and effects of mental retirement, there are indications that it might have negative consequences for employees (e.g. lower job satisfaction and mental health), and therefore also for the organization (reduced productivity, absenteeism, profit loss) and society in general (costs due to early retirement, well-being) [3, 9, 13, 28, 29]. Thus, it appears important that mental retirement among workers is prevented or reduced as much as possible. Therefore, an organizational program was developed to reduce mental retirement.

### Important features of an organizational program in general

Research has shown there are several features that are important in order for organizational programs or interventions to be effective. First, a tailor-made approach is necessary. Tailoring improves the utilization of the results, helps to choose the interventions that meet the specific needs of the team or organization and make effective action plans [30–32]. However, this approach is not easy to implement or evaluate since it means that it is impossible to develop a single intervention that will be effective in every organization or team. Second, previous research has also shown that a stepwise approach is effective by providing a framework for organizations that can be used to make suitable choices for interventions [31]. This stepwise approach often consists of five basic steps that are specified for each program: 1) raising awareness and creating commitment in an organization, 2) problem assessment, 3) prioritizing, choosing interventions and making an action plan, 4) implementation of the chosen intervention(s), and 5) evaluation of the process and effects [30, 33]. A third important feature is a participatory approach. Research has shown that the use of participatory action research is very successful for occupational health interventions [31, 33–35]. By using the knowledge, skills and perceptions within the team (of the employees as well as the supervisors/management), a feeling of joint ownership is created during the program. Participants feel more in control and experience a greater sense of fairness and justice. Thereby, not only is awareness created but also a joint responsibility for both the problems as well as the solutions for these problems. In addition, the participation can also decrease the resistance to change and smoothen the change process [33, 36, 37].

Next to the features of an organizational program the research design is also very important, especially in applied research. Although a randomized controlled trial (RCT) is often considered as the golden standard, this is not always feasible. Particularly in applied occupational health research, the interventions and the context are often complex and therefore hard to control [38–41]. Therefore, in applied research quasi-experiments with a control and intervention group are often difficult to establish and complete in a satisfactory way. Additionally, even if a RCT is performed with success, the question remains whether the conclusions can be generalized to other organizations and individuals or that the results only apply for a selected sample of individuals [40, 42]. Using process evaluation and incorporating the measurement of intervention exposure in participants’ samples is an alternative way to cope with these problems [38, 41]. Data on exposure to the intervention can easily be obtained in an intervention process evaluation by asking participants about their experiences and exposure. This information can then be used to determine whether each participant is more appropriately placed in an intervention/exposed group or a control/not exposed group. This approach makes it possible to take into account the complex, chaotic and uncontrollable organizational settings.

### The present study: The design of the mental retirement program

**Fig. 1:**
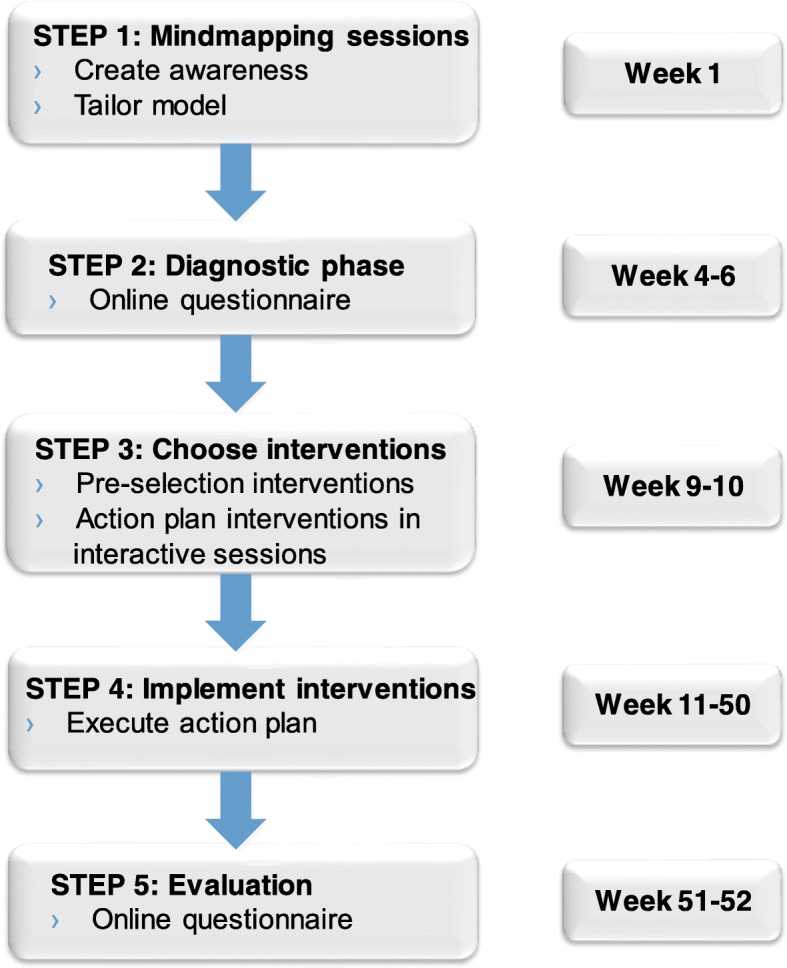
Mental retirement program

To address the issue of mental retirement, a bottom-up program was designed based on the principles of a participatory, stepwise, tailor-made approach. The program consists of five steps (see Fig. 1). The program is carried out by the whole team, i.e., employees and supervisors together. In the first step, a representative delegation of the team participates in *mindmapping sessions*. Two sessions are held with 10–20 participants each. These sessions are each led by two facilitators. At least one of the facilitators is a researcher, the other can also be a researcher or an employee of the organization that is trained in the mental retirement program. The sessions have two goals. First, these sessions create awareness of mental retirement within the team and gain acceptance for the program and the possible changes it may bring. Secondly, the information gained during the sessions is used to tailor the model of mental retirement with organization-specific concepts and to adjust the basic questionnaire accordingly. In the mindmapping sessions the employees and supervisors discuss what they believe mental retirement is and what the predictors and effects are of mental retirement. To not only create awareness but to also set people in motion, the participants contemplate on possible actions for themselves, their supervisor and the organization to decrease mental retirement. The employees who participate in the mindmapping sessions are contacted by management or the HR-professional of the department who invite them to participate. Participation is voluntary. The employees that are approached are selected so that they represent the entire department with regard to age, sex, function, time employed and level of mental retirement (as estimated by the manager or HR-professional). Furthermore, in this stage a project group of approximately six people is created, which consists of several employees of the team, a HR-professional and sometimes the supervisor or manager. The team itself decides who will join this project group and participation is voluntary. The project group is the first point of contact for the researchers as well as for all the employees in the department.

In the *diagnostic phase*, the basic model of mental retirement is tailored to the team with the input of the mindmapping sessions. Where necessary questions are added to the basic questionnaire. This questionnaire is available in an online portal for all the team members to fill out.

In a tailor-made intervention process, step 3, the team *chooses the interventions* that they want to implement in order to decrease mental retirement in their team. Each team chooses their own intervention(s), so these may differ between teams. The interventions are selected based on the results of the baseline questionnaire as well as their fit with the team in terms of process and culture. First a pre-selection is made by the project group in one session. Next, the team results of the baseline questionnaire and the pre-selection of interventions are presented in two interactive sessions. In these sessions the team members formulate an action plan for the implementation of the interventions they agree with. When necessary, they select or design new interventions. The participants in these sessions are the same as those who participated in the mindmapping sessions. The results of the questionnaire and the action plan are distributed across the rest of the team in a way the project group sees fit.

In the next step, the organization is in charge and the role of the researchers is marginalized. The team starts to *implement the interventions*. The selected interventions do not necessarily start all at once, but are spread out through time. There needs to be enough time for carrying out the action plan and for the interventions to take place and have effect. Therefore, the duration of this implementation step takes approximately six to nine months.

In the fifth and final step, the program is *evaluated*. In consultation with the project group and based on the implementation process of the interventions, the timing of the follow-up questionnaire is determined. Approximately one year after the start of the intervention (step 1), an online follow-up questionnaire is made available in the portal to evaluate the effects of the program. The questionnaire is largely the same as the baseline questionnaire, but also includes questions regarding the interventions that were implemented.

The current study aims to explore the effects of this mental retirement program in a broad population of employees within several organizations. The effect of the program as a whole is examined, rather than the effects of the specific interventions that are implemented in step 4 of the program. As each team or department chooses their own interventions, the range of interventions is very wide which makes it difficult to examine the effects of each intervention separately. In addition, we believe that the effect of our program is due to the program as a whole (all 5 steps) and not so much to the specific interventions of step 4.

- Hypothesis 1: the level of mental retirement will decrease between baseline and follow-up, due to the mental retirement program as a whole and independent of the specific interventions that are implemented in step 4.
- Hypothesis 2: the improvement in mental retirement will be stronger for employees who actively participated in the interventions than for those who participated to a lesser degree.

## Methods

### Design and procedure

The research population of this study consisted of a sample of employees of five departments of three different organizations in the Netherlands. Three departments of the Dutch National Police participated: two departments of police officers and one facility department (*N* = 175; *N* = 185; *N* = 175, respectively). The other two organizations were an archive department of the Dutch government (*N* = 291) and one location of a non-profit organization that implements national insurance schemes in the Netherlands (*N* = 209). The reasons for participating in this study differed across organizations and teams, but included issues such as minimizing the effects of a reorganization, creating more awareness among employees regarding their own development, getting employees out of ‘sleep mode’ to prevent them from getting stuck in their career.

Online questionnaires were sent to every employee of all departments (*N* = 1035) in step 2 (baseline measurement) and step 5 (evaluation). The baseline questionnaires were sent between March 2014 and October 2015 (depending on when the organization started with the program). The follow-up questionnaire was sent out approximately one year after the baseline questionnaire.

### Measures

Mental retirement was measured with three concepts [8]. Firstly, *developmental pro-activity* consisted of four items [43]. An example item is: “I think about how I can keep doing a good job in the future”. The response categories ranged from 1 (“completely disagree”) to 5 (“completely agree”). The internal consistency in our study (Cronbach’s alpha) was .85. Secondly, *work engagement* was measured with six items (three items on vigor and three items on dedication) from the Utrecht Work Engagement Scale (UWES) [44], including “My job inspires me” (α = .94). Respondents were asked to describe how often they experienced the described situations (1 = never; 7 = always). Thirdly, *perceived appreciation*, is measured with one question: “Do you feel appreciated in your current job?”. The response categories ranged from 1 (“not at all”) to 4 (“very much”). The scale scores of the three mental retirement sub-concepts were standardized (into scores between 0 and 1) because their response categories differed. A mean score was calculated over these three sub-concepts, which created a variable that measured mental retirement in one variable (range 0 to 1; α = .64).

In the follow-up questionnaire measures of *intervention exposure* were included. Respondents were asked if they were familiar with each intervention (0 = “no”, 1 = “yes”) and if so, to what extent they had participated in the specific intervention(s) which were chosen and implemented at their workplace (1 = “not”, 5 = “very much”). For each respondent the intervention exposure was calculated: a sum score was made of the number of interventions in which they had participated (very) much, divided by the maximum number of interventions they could participate in.

### Statistical analysis

Missing values were imputed using multiple imputation procedures in the SPSS “Missing Values” module, based on an iterative Markov chain Monte Carlo (MCMC) method. Overall data missingness was 31.5%, mainly due to dropout at follow-up and not filling out the questionnaires completely. It has been shown that multiple imputation-based procedures are superior to case-wise deletion of missing data [45]. In our analyses, only data were used that did not have imputed data on the variable intervention exposure. The resulting data set comprised 466 members of five departments.

Multilevel modelling (MLM) [46] was used to study our hypotheses and analyze our data, which were nested at the organizational level. The MLM analyses were performed in SPSS version 25.0 Multilevel modelling (i.e. hierarchical linear model) which aims to analyze data that contains an inherent hierarchical structure [47]. In the present study the data contains two levels. The first of lowest level of the data contains individual scores of mental retirement at baseline and follow-up (within-subject level). At the second level the individuals are nested into departments (between departments). In a stepwise procedure a final model was built for each outcome. First, the presence of a random intercept was tested for each outcome, indicating whether departments have different intercepts. In the second step, the presence of a random slope was tested for each outcome measure, indicating whether departments differed in the way their mental retirement changes over time. In the final step, educational level was added as a covariate to the best-fitting model.^1^ For the first hypothesis, difference scores (between baseline and follow-up) were calculated for each outcome and used as dependent variable, making the intercept of the model an indicator for the change in the outcome from baseline to follow-up. For the second hypothesis, the dependent variable was the follow-up measurement. In the final step of the analysis, the baseline measurement of the outcome and the intervention exposure (the extent employees had participated in the specific intervention(s) in their team) were added. Variables in the equation were not centered, because all included variables had interpretable zero values. The intraclass correlation coefficient (ICC) was calculated to obtain the amount of variance explained by the differences between departments.

Besides the quantitative results, also qualitative results are reported. The researchers monitored the context and setting of each department and kept track in a digital logbook. In the logbook, the sequence of planned and unplanned events was listed alongside impressions of the researchers. These impressions were based on their own observations during the sessions. At the end of each session the researchers shortly evaluated the sessions by asking the opinion of the participants. In addition, the impressions of the researchers are based on their periodical contacts with the project group. In these contacts, the project group informed the researchers on their observations as well as the progress made. Furthermore, the researchers logged all changes that occurred in the context or setting of the organizations. The logbook was kept up to date throughout the duration of the study. The logbook data were grouped per department to form a chronological list of events, including the impressions of the researchers. The qualitative results will be discussed in accordance with the five steps of the program.

## Results

In total 683 (66.0%) employees filled out (part of) the baseline questionnaire and just over 400 (39.5%) participants filled out (part of) both questionnaires (see Table 1 for response-rates within each department).^2^ The mean age of the participants was 45.8 years (see Table 2) and most were male (60.3%) and had an intermediate level of education (50.6%). Most participants worked fulltime (69.6%) and had been working on average almost 13 years within their current organization. Table 2 also displays the scores on mental retirement and its sub-concepts at baseline.

**Table 1:** Response-rate within each department

Department	Number of employees	Number of respondents at baseline (%)	Number of respondents at follow-up (%)	Number of respondents overall (%)^a^
Police officers department 1	175	128 (73.1)	121 (69.1)	86 (49.1)
Police officers department 2	185	102 (55.1)	54 (29.2)	32 (17.3)
Facility department police	175	102 (58.3)	73 (41.7)	49 (28.0)
Archive department	291	196 (67.4)	194 (66.7)	141 (48.5)
National insurance schemes department	209	155 (74.2)	128 (61.2)	101 (48.3)
Total	1035	683 (66.0)	570 (55.1)	409 (39.5)

^a^based on employees who filled out (part of) both questionnaires

**Table 2:** Baseline characteristics of the participants

Variable		Percentage or Mean/SD
Gender	Male	60.3%
(*N* = 667)	Female	39.7%
Education	Lower	26.1%
(*N* = 666)	Intermediate	50.6%
	Higher	23.3%
Working hours per week	> = 35 h	69.6%
(*N* = 667)	20–34 h	28.0%
	12–19 h	1.3%
	< 12 h	1.0%
Age	Mean	45.8
(*N* = 654)	Standard Deviation	11.4
Years working at organization	Mean	12.8
(*N* = 683)	Standard Deviation	10.9
Years working in job	Mean	6.7
(*N* = 683)	Standard Deviation	6.9
Mental retirement	Mean	.36
(*N* = 403)	Standard Deviation	.16
Developmental pro-activity	Mean	4.12
(*N* = 409)	Standard Deviation	.59
Work engagement	Mean	4.83
(*N* = 403)	Standard Deviation	1.33
Perceived appreciation	Mean	2.46
(*N* = 403)	Standard Deviation	.78
Intervention exposure	Mean	.19
(*N* = 466)	Standard Deviation	.25

### Quantitative results

Table 3 displays the effect of the program on mental retirement and its sub-concepts. Since none of the intercepts are significant, the level of mental retirement does not change between baseline and follow-up. Therefore, hypothesis 1 is rejected.

**Table 3:** Effect of the program on the difference scores of mental retirement and its sub-concepts (*N* = 466)

	Mental retirement B (95% CI)	Developmental pro-activity B (95% CI)	Work engagement B (95% CI)	Perceived appreciation B (95% CI)
Intercept	.00 (−.04–.04)	−.05 (−.20–.11)	.10 (−.16–.36)	−.01 (−.21–.20)
Lower education	−.01 (−.05–.04)	−.06 (−.25–.13)	−.05–.37–.27)	.12 (−.14–.37)
Intermediate education	−.01 (−.04–.03)	.06 (−.09–.21)	−.00 (−.26–.26)	.03 (−.18–.24)
ICC	.03	.03	.02	.02

In Table 4 the results of the multilevel analyses that take the level of intervention exposure into account are shown for each outcome. There is a significant effect of intervention exposure on mental retirement. Employees who were more exposed to the intervention(s) (i.e. who more often participated (very) much in the intervention(s)), had a slightly lower level of mental retirement at follow-up. This effect was also found for two of the sub-concepts of mental retirement; developmental pro-activity and work engagement. For perceived appreciation only a tendency was found. These results show that active participation in the intervention(s) is related to a decrease of mental retirement, which is in line with hypothesis 2.

**Table 4:** Effect of the program on mental retirement and its sub-concepts at follow-up, factoring in the level of intervention exposure (*N* = 466)

	Mental retirement B (95% CI)	Developmental pro-activity B (95% CI)	Work engagement B (95% CI)	Perceived appreciation B (95% CI)
Intercept	.17** (.12–.21)	2.50** (2.10–2.90)	1.86** (1.39–2.34)	1.41** (1.10–1.71)
Baseline^#^	.54** (.46–.61)	.39** (.30–.48)	.62** (.55–.70)	.44** (.35–.53)
Intervention exposure	−.07** (−.11 – −.02)	.24* (.05–.42)	.39* (.05–.74)	.25^†^ (−.02–.52)
Lower education	-.02 (−.06–.02)	−.12 (−.28–.04)	.16–.14–.46)	.10 (−.12–.32)
Intermediate education	.00 (−.03–.03)	.01 (−.11–.14)	.04 (−.19–.27)	−.07 (−.25–.11)
ICC	.06	.02	.11	.04

^†^(*p* < 0,10), *(*p* < 0,05), **(*p* < 0,01)

^#^Baseline measurement of the outcome measure

### Qualitative results

The *mindmapping sessions* did play an important role in increasing the enthusiasm of the employees. The participants valued the possibility of not only giving their opinions, but also that these were taken seriously and that they had a say in the following steps of the program. Although the mindmapping sessions were valued, the participants often had trouble to make things specific, especially when contemplating on possible actions to decrease mental retirement. Therefore, it was important that the facilitators dug deeper and asked more questions.

The way participants received the results of questionnaires during the *diagnostic phase* changed throughout the study. Halfway through the study the questionnaires had to be administered in a new digital portal. In this new portal participants received their results directly after finishing their questionnaire. This personal report not only included their personal results, but also tips and feedback about how to improve their scores. This new portal was implemented in two of the teams within the police force. The participants of the other two organizations and the third team of the police filled out the questionnaires in the ‘old’ digital environment, which did not have a personal report and direct feedback. The content of the questionnaire was the same in all teams (of course except for the tailored questions). Another obstacle in this phase was the timing of the questionnaire. Sometimes the questionnaire had to be sent out during the same time as the employee engagement survey or a survey for a different study. This had possible negative effects on the response-rate. However, the timing of the questionnaire was always in coordination with the project group.

In the interactive sessions where the participants *choose the interventions* that would be implemented and made an action plan for this implementation, the participants again valued the possibility to give their opinion and the influence they had on the implementation-plan. The participants were perfectly able to make up their mind about the suggested interventions, to tailor those interventions for their own team or organization and to make an action plan. However, it was difficult for them to come up with new interventions themselves based on the results of the questionnaire and their own needs. Examples of interventions that were implemented in the teams are making employees themselves responsible for the distribution of work; allowing every employee to spend two hours per week on keeping their knowledge up-to-date; “secret service” (i.e. employees are rewarded and praised for small and big accomplishments without knowing who gave them the reward or praise); and various training programs, including a training in ‘appreciative dialog’ (which is based on the appreciative inquiry aiming at enforcing the positive instead of battling the negative); a training in providing feedback; a training on the job (by giving employees more opportunities for informal learning during their normal work tasks); and a training in job crafting.

In the *intervention implementation phase*, the teams struggled to effectively implement the interventions that were chosen and to keep the team members involved in the program. Nevertheless, the program still continued and in all teams interventions were implemented. However, in some cases these were different interventions than initially planned, due to evolving circumstances and insights gained. One of the teams installed a project manager whose fulltime job it was to implement the action plan and the interventions. This helped to keep the focus on the program and to carry out the action plan.

Carrying out the effect *evaluation* and sending out the second questionnaire was difficult. The response rate was lower (see Table 1), mostly due to the decreased focus on the program as is described above. In addition, due to the restructuring in one organization the team members changed during the study. Employees transferred to other teams that didn’t participate in the program and employees from other teams started working in a team that did participate. Of course in all organizations there were also some changes in team members because employees retired, got a new job and new employees were hired, but these numbers are quite low.

With regard to the *overall context and setting* of the organizations, several factors had an influence. In one organization a restructuring took place during the study, in another organization that operates in a political environment there was a change in responsibilities for a national insurance and in the last organization the study started just after a new director was assigned. All these changes started before the beginning of the study and the organizations deliberately chose to still start with the mental retirement program because especially in such situations it is important to take control over your own development.

## Discussion

The present study investigated the effect of a stepwise, bottom-up participatory program to decrease the level of mental retirement of employees in three different organizations. This study showed no difference in mental retirement between baseline and follow-up one year later. However, multilevel analysis also showed that employees who actively participated in the intervention(s) that were implemented during step 4 of the program did show a decrease in mental retirement and its sub-concepts. By incorporating the measurement of intervention exposure, a type-III error (incorrectly concluding that an intervention is ineffective when it is actually its *implementation* that is suboptimal) was prevented.

An important aspect of the mental retirement program is its bottom-up participatory design. In all steps of the program the whole team is involved and together they decide what interventions will be implemented and how to do this (by making an action plan). The positive effects of the use of participatory designs have been well established in other studies [31, 33–35]. Such designs can lead to feelings of joint ownership, control and responsibility, a greater sense of fairness and may smoothen the change process [33, 36, 37]. A second important feature of the mental retirement program is the stepwise, tailor-made intervention process. This approach increases the chance that the interventions that are chosen meet the specific needs of the team better, that the action plans that are made are more effective and that the results are better used [30–32]. These two features of the mental retirement program (participatory design and the stepwise, tailor-made intervention process) might be more important in explaining the effects that were found in the present study than the specific interventions that were implemented in each team during step 4 of the program.

Even though the present study shows small but good effects of the mental retirement program, there are some improvements imaginable. First of all, in the current study the setting and context of the organizations changed throughout the study. Although the researchers kept a log book to document these changes, it is difficult to pinpoint if and how these changes affected the results. In addition, the response-rate on baseline and follow-up was reasonable, but the number of employees that filled out (part of) both questionnaires was low in some departments. Furthermore, in the intervention implementation phase, the organization is in the lead and has complete autonomy and the role of the researchers is marginalized. During this study it appeared that this might be too big a change compared with the first three steps of the program. The teams struggled to keep the program ‘alive’, to preserve the commitment of the team members and to implement changes when there were no researchers to keep them on track. Earlier research has also shown this struggle [48, 49]. One of the departments solved this problem by installing a fulltime project manager whose job it was to implement the action plan and the interventions. So, although a participatory design is important to create commitment and ownership, it appears that there has to be some guidance or coaching.

### Strengths and limitations

To our knowledge, the present study is the first that examines the effects of a program for diminishing mental retirement. Such a program may become more important because of the increasing interest in sustainable employability due to the rapid changes in skills demands and the labor market and the fast aging of the workforce. Therefore, there is a necessity for more attention and awareness for concepts like mental retirement. Another strength of this study is that this study shows that more generic principles like a participatory, stepwise and tailor-made approach, appear to be more important than the specific interventions that are implemented within an organization. Future research should focus more on these generic principles and study how and in what circumstances this leads to success.

However, when interpreting the findings of this study some limitations should be kept in mind. First, although an intervention exposure measure was used in this study a control group is missing. Even employees who reported that they had not actively participated in the intervention(s), are exposed to the program. All the members of a department were informed about the mindmapping sessions, the results of the baseline questionnaire and the interactive sessions where plans of actions were made for the intervention(s). In addition, only employees that reported to participate (very) much in an intervention were classified as exposed to an intervention. Employees that reported to participate only a little in an intervention were classified as non-exposure. So, in the present study it wasn’t possible to select a control group that wasn’t exposed to the mental retirement program. Second, in this applied research study the contextual setting changed continuously, for example by the restructuring in one organization. It is possible that confounding biased and influenced the data [50]. To get more grip on the contextual setting, the researchers kept track of changes to the setting in a logbook. Furthermore, multilevel modelling was used to correct the cluster effect. Third, in the present study only two questionnaires were sent out, one at baseline (after the mindmapping session) and one at follow-up (approximately one year later). It would be interesting to see what the effects of the program are when using more measurements, for instance a baseline measurement before the mindmapping sessions and follow-up measurements both on short term as well as long term. Especially since in the present study the process of implementation of the interventions was a challenge and commitment to the program was possibly lost during this phase. Therefore, future research should be longitudinal in nature, have multiple measuring moments to look into both short term as well as long term effects and have a more extensive process evaluation. Furthermore, new research should focus more on exploring the concept of mental retirement itself and also explore the predictors and the effects of mental retirement. Also, other sources of data should be considered since the present study only makes use of self-report which can be prone to recall bias. Last, new studies should use a more powerful manipulation of the groups to see what the effects are of the mental retirement program in a study with a control group with employees that have no knowledge of the mental retirement program at all, compared to a intervention group that did participate in the program.

## Conclusions

The present study aimed to gain insight in the effect of a bottom-up participatory program to decrease the level of mental retirement of Dutch employees. This study showed that the participatory program had positive effects: it tends to decrease the level of mental retirement for employees who actively participated in the intervention(s) that were implemented during step 4 of the program. Important aspects of the mental retirement program are a bottom-up participatory approach and a stepwise, tailor-made intervention process. However, the phase of implementing the interventions could be further improved since this process proved to be very challenging and commitment to the program was diminished during this phase. Although the present study showed small effects and had some limitations in design, future research could study the effectiveness of this program further to strengthen the concept of mental retirement. Future research should not only study the concept itself but also its predictors and make use of an improved design with for instance a control group, multiple follow-ups and several data sources.

## Abbreviatons

HR: Human Resources
ICC: Intraclass correlation coefficient
MCMC: Markov chain Monte Carlo
MLM: Multilevel modelling
RCT: Randomized controlled trial
UWES: Utrecht Work Engagement Scale

## Footnotes

1 A model of random intercepts was best-fitting for all outcomes. A random slopes model did not fit any outcome variable best.

2 No significant differences on baseline levels of mental retirement and its components were found between drop-outs (only filled out the baseline questionnaire) and employees who filled out both questionnaires.
